# Use of the mice passive protection test to evaluate the humoral response in goats vaccinated with Sterne 34F2 live spore vaccine

**DOI:** 10.1186/s13567-017-0451-4

**Published:** 2017-09-07

**Authors:** P. H. Phaswana, O. C. Ndumnego, S. M. Koehler, W. Beyer, J. E. Crafford, H. van Heerden

**Affiliations:** 1 Department of Veterinary Tropical Diseases, University of Pretoria, Onderstepoort, 0110 South Africa; 2 Department of Livestock Infectiology and Environmental Hygiene, Institute of Animal Science, University of Hohenheim, Emil-Wolff-Strasse 14, 70599 Stuttgart, Germany; 3 Africa Health Research Institute, K-RITH Tower Building, Umbilo Road, Durban, 4013 South Africa; 4 Present Address: Robert Koch Institute, Nordufer 20, 13353 Berlin, Germany

## Abstract

The Sterne live spore vaccine (34F2) is the most widely used veterinary vaccine against anthrax in animals. Antibody responses to several antigens of *Bacillus anthracis* have been described with a large focus on those against protective antigen (PA). The focus of this study was to evaluate the protective humoral immune response induced by the live spore anthrax vaccine in goats. Boer goats vaccinated twice (week 0 and week 12) with the Sterne live spore vaccine and naive goats were used to monitor the anti-PA and toxin neutralizing antibodies at week 4 and week 17 (after the second vaccine dose) post vaccination. A/J mice were passively immunized with different dilutions of sera from immune and naive goats and then challenged with spores of *B. anthracis* strain 34F2 to determine the protective capacity of the goat sera. The goat anti-PA ELISA titres indicated significant sero-conversion at week 17 after the second doses of vaccine (*p* = 0.009). Mice receiving undiluted sera from goats given two doses of vaccine (twice immunized) showed the highest protection (86%) with only 20% of mice receiving 1:1000 diluted sera surviving lethal challenge. The in vitro toxin neutralization assay (TNA) titres correlated to protection of passively immunized A/J mice against lethal infection with the vaccine strain Sterne 34F2 spores using immune goat sera up to a 1:10 dilution (r_s_ ≥ 0.522, *p* = 0.046). This study suggests that the passive mouse protection model could be potentially used to evaluate the protective immune response in livestock animals vaccinated with the current live vaccine and new vaccines.

## Introduction

Anthrax is a zoonotic disease caused by *Bacillus anthracis* that affects all mammals especially herbivorous mammals [1]. The main virulence factors are encoded by two plasmids, pXO1 and pXO2 [2]. The pXO1 encodes for the tripartite protein exotoxin (anthrax toxin) that consists of the cell-binding protective antigen (PA), and two enzymes known as lethal factor (LF) and edema factor (EF) [3]. The pXO2 encodes for the anti-phagocytic poly-D glutamic acid (PGA) capsule which protects the bacteria against phagocytosis [4].

Lethal toxin (LT) is formed by the binding of LF to PA while edema toxin (ET) formation occurs when EF binds to PA [2, 3]. The highly immunogenic PA therefore plays a central role in the activation of both LT and ET and was shown to elicit a protective immune response against anthrax in both experimental animals and humans as reviewed by Little et al. [5]. Virulence of *B. anthracis* is also dependent on the presence of the anti-phagocytic capsule and the absence of either pXO1 or pXO2 will result in attenuation of the organism [6].

The current anthrax veterinary vaccine comprises of spores from the live, attenuated *B. anthracis* 34F2 strain developed in 1937 by Max Sterne and known as the Sterne live spore vaccine [7]. The vaccine is a stable uncapsulated mutant that produces all three toxin components of *B. anthracis* (PA, LF and EF) whilst still providing protective antigens [7, 8]. Humoral immunity develops 2–4 weeks after first vaccination and revaccination is recommended every 9–12 months (Anthrax vaccine leaflet, Onderstepoort Biological Products, South Africa, http://www.obpvaccines.co.za) [9]. In vivo assessment of the Sterne vaccine immunity mainly involved pathogenicity and efficacy testing during which guinea pigs were vaccinated with Sterne live spore vaccine and challenged with *B. anthracis* strain 17 JB (Pasteur II strain) [9]. The use of serological techniques for the detection of correlates of protection to anthrax vaccine was initiated with the search for efficacious human vaccines.

Until recently the few studies on the immune response induced by the Sterne live spore vaccine in ruminants were done by Turnbull et al. [10] and Shakya et al. [11]. Ndumnego and Kohler et al. [12] vaccinated Boer goats with one or two doses of Sterne 34F2 live spore vaccine and challenged them with fully virulent *B. anthracis* spores. All the goats receiving two vaccine doses survived virulent challenge whereas 60 and 80% of those vaccinated once survived. Sera collected post-challenge from these animals were used as the positive controls in this study. In this report, we evaluated the humoral immune response induced by the live spore anthrax vaccine in Boer goats using the anti-PA ELISA, an in vitro toxin neutralization assay (TNA) and a passive protection test in A/J-mice as first performed with large wild animals by Turnbull et al. [13].

## Materials and methods

### Sterne live spore vaccine

The Sterne live spore vaccine is produced by Onderstepoort Biological Products (OBP) in South Africa and consists of *B. anthracis* strain 34F2 (pXO1^+^, pXO2^−^) spores suspended in glycerine at a spore concentration of 6 × 10^6^ per mL.

### Experimental animals

Healthy, age-matched female and emasculated male naive Boer goats were used for production of immune sera. These animals were sourced from livestock farms in Pretoria area (Gauteng Province, South Africa), confirmed to be PA-reactive antibody negative by ELISA and kept at the OBP experimental animal facility. The animals were fed pellets and hay with water ad libitum. They were dewormed following arrival and kept in a fenced, outdoor facility with concrete floors throughout the trial. All goats were kept together for the entire period of the experiment.

Six to eight weeks old female A/J mice were used in the passive protection test. A/J mice are deficient for the C5 complement component rendering them highly susceptible to the Sterne live spore vaccine strain [14]. These mice were obtained from Jackson Laboratories, USA, and maintained in pathogen-free conditions at the laboratory animal facility of the University of Pretoria Biomedical Research Centre (UPBRC) according to the South African national standard for the care and use of animals for scientific purpose. All studies were approved by the animal ethics committee (AEC) of the University of Pretoria, South Africa (protocol approval number V083/13) and permission was granted (12/11/1/1) by the Department of Agriculture, Forestry and Fisheries, South Africa under the animals disease act (Act 35 Section 20, 1984).

### Vaccination regimen

The experimental group consisted of Boer goats (*n* = 5), immunized twice with the Sterne live spore vaccine (6 × 10^6^ per mL). One ml OBP Sterne vaccine was administered subcutaneously at week 0 and week 12 according to the manufacturer’s instruction. A negative control group of Boer goats (*n* = 3) received only sterile saline (1 mL) instead of vaccine. The animals were bled before the initial vaccination (week 0) and at week 4 and week 17. Positive control sera (*n* = 4) were sourced from Boer goats which survived challenge with virulent anthrax spores after two vaccinations with the Sterne live spore vaccine in a previous study [12].

### Anti-PA antibody ELISA

Sera collected at weeks 0, 4 and 17 were analysed for PA-specific antibody using the ELISA as previously described by Hahn et al. [15] and Pombo et al. [16] with some modifications as described by Ndumnego et al. [17]. Endpoint titres of individual serum were defined as the reciprocal of the highest serum dilution giving an optical density of 0.1. Titres of < 50 were ascribed an arbitrary value of 0. Sera from immune goats (*n* = 4) that survived a virulent *B. anthracis* challenge and naive goats (*n* = 3) were used as positive and negative controls respectively.

### Toxin neutralization assay (TNA)

An in vitro toxin neutralization assay (TNA) was performed using a MTT [3-(4.5-dimethylthiazol-2-yl)–2.5-diphenyltetrazolium bromide] in a colorimetric cell viability assay with the J774A.1 macrophage cell line as previously described by Hering et al. [18] with slight modifications by Ndumnego et al. [17]. Briefly, serial diluted goat serum was incubated with PA and LF (List Biological Laboratories, USA) at concentrations of 500 and 400 ng/mL respectively before addition to overnight incubated macrophage cells. The following controls were added to each assay: a single dilution series of positive serum from a Sterne hyper-immunized goat which survived virulent challenge served as positive control [12], three wells (without cells) with medium and LT served as blanks, three wells (with cells) and LT served as toxin control and two wells with cells and culture media served as medium control. Cell viability was determined by reading the optical density (OD) at 540 nm after the addition of the MTT tetrazolium dye. The neutralization titre of each serum was calculated by NT_50_ = (sample OD − toxin control OD)/(medium control OD – toxin control OD) × 100 and expressed as the reciprocal of the highest serum dilution neutralizing 50% of the toxins.

### Passive transfer of serum and challenge in A/J mice

A/J mice (*n* = 96) were used in the passive protection tests as previously described by Turnbull et al. [13]. Briefly, serum from twice immunized individual goats (*n* = 5), collected on week 17, were used either undiluted or diluted in physiological saline (1:1000, 1:100, 1:50, 1:10, 1:5). Each of these dilutions were administered intraperitoneally (500 µL) to three A/J mice (*n* = 3 for each goat serum) for each dilution from individual goats (*n* = 15). Positive control mice (*n* = 12, three A/J mice for each goat serum) received undiluted sera from immune goats (n = 4) that survived a virulent *B. anthracis* challenge. Negative control mice (*n* = 9, three A/J mice for each goat serum) received undiluted sera from naïve goats (*n* = 3) as performed previously [13]. The mice were challenged after 24 h by subcutaneous inoculation with 1.92 × 10^5^ spores [13] from Sterne 34F2 vaccine strain and monitored for survival over 14 days. Death due to anthrax was confirmed by re-isolation of *B. anthracis* from liver and spleen smear cultures on sheep blood agar. Survivors were euthanized by injecting a barbiturate overdose and confirmed free of Sterne spores following culture of liver and spleen.

### Statistics

We compared the immune response in Sterne vaccinated goats using ELISA and TNA with the passive protection test in mice. The relationship between individual ELISA, TNA antibody titres and time of survival was described using the Pearson’s correlation coefficient. Differences in antibody titres (ELISA and TNA) between points of measurement were analysed using a two-tailed (paired) Student *t* test. *p* values of < 0.05 were considered statistically significant. The survival times for mice receiving different goat serum dilutions were analysed using the Kaplan–Meier method in SPSS Version 21 (IBM SPSS Statistics, USA). Log-rank test was used to compare the survival curves of test and control groups. The anti-PA and TNA linear plot was done using Sigma Plot (Systat software Inc, USA).

## Results

### Anti-PA antibody ELISA and TNA

**Figure 1:**
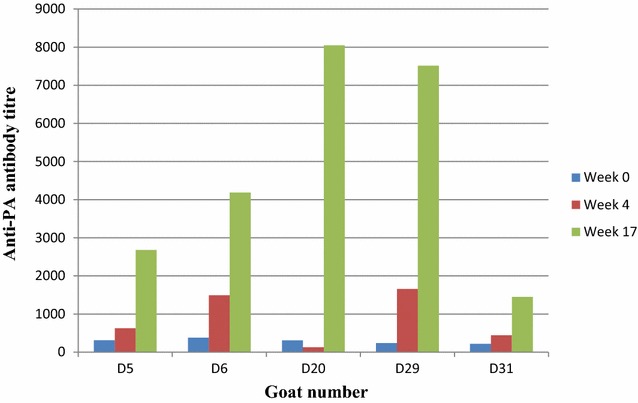
**Anti-protective antigen (PA) antibody titres as detected by ELISA following Sterne live spore vaccination of goats at week 0 and week 12.** Serum samples of individual goats (D5, D6, D20, D29 and D31) were collected and analysed before vaccination (week 0), 4 weeks after initial vaccination (week 4) and after the second vaccine dose (week 17). Mean anti-PA antibody titres detected by ELISA of positive control (Sterne live spore vaccinated goats) and negative control (naive goats) are indicated in Table 1.

ELISA was performed to determine the serum IgG antibody levels against PA from individual sera collected at weeks 0, 4 and 17. Serum anti-PA antibody titres were very low in naïve animals, both the animals before the first vaccination and control animals injected with sterile saline. Titers in the latter group remained low until the end of the experiment. The anti-PA titres of each individual animal before vaccination (week 0), after first vaccination (week 4) and after second vaccination (week 17) are indicated in Figure 1. There was no significant difference in mean titres between week 0 and 4 (*p* = 0.0913) but there were significant differences between week 0 and 17 (*p* = 0.009) and between week 4 and 17 (*p* = 0.019). The goat anti-PA ELISA titres indicated significant sero-conversion at week 17 after the second doses of vaccine (*p* = 0.009).

**Figure 2:**
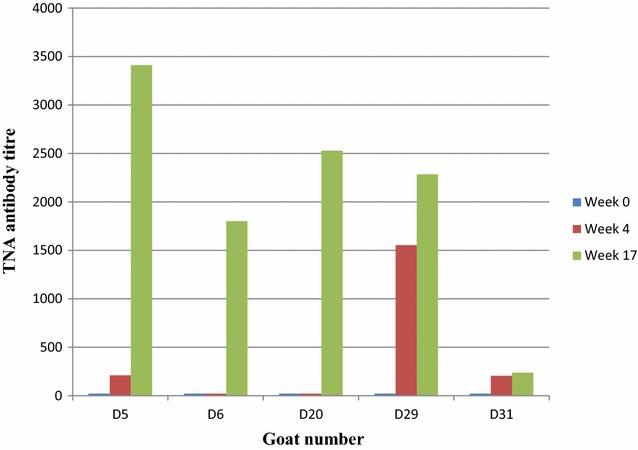
**Toxin neutralization antibody titres following Sterne live spore vaccination of goats at week 0 and week 12.** Serum samples of individual goats (D5, D6, D20, D29 and D31) were collected and analysed before vaccination (week 0), 4 weeks after initial vaccination (week 4) and after the second vaccine dose (week 17). Mean toxin neutralization antibody titres of positive control (Sterne live spore vaccinated goats) and negative control (naive goats) are indicated in Table 1.

An in vitro TNA was performed to assess toxin neutralizing antibodies in sera of vaccinated goats using murine J774A.1 macrophage cell line. Neutralizing antibody titres measured by the TNA assay varied among the individual animals. There were no detectable neutralizing titres in sera collected at week 0 (before vaccination). The TNA titres at week 4 were low and goat D6 and D20 did not give neutralization titres after the first vaccination. All five immune goat sera showed neutralizing activity at week 17 after the second vaccine dose (Figure 2). No significant differences were found between TNA titres at week 0 and 4 (*p* = 0.218), but there was significant difference in mean titres between week 0 and 17 (*p* = 0.004) and between week 4 and 17 (*p* = 0.025).

### Passive protection test

**Figure 3:**
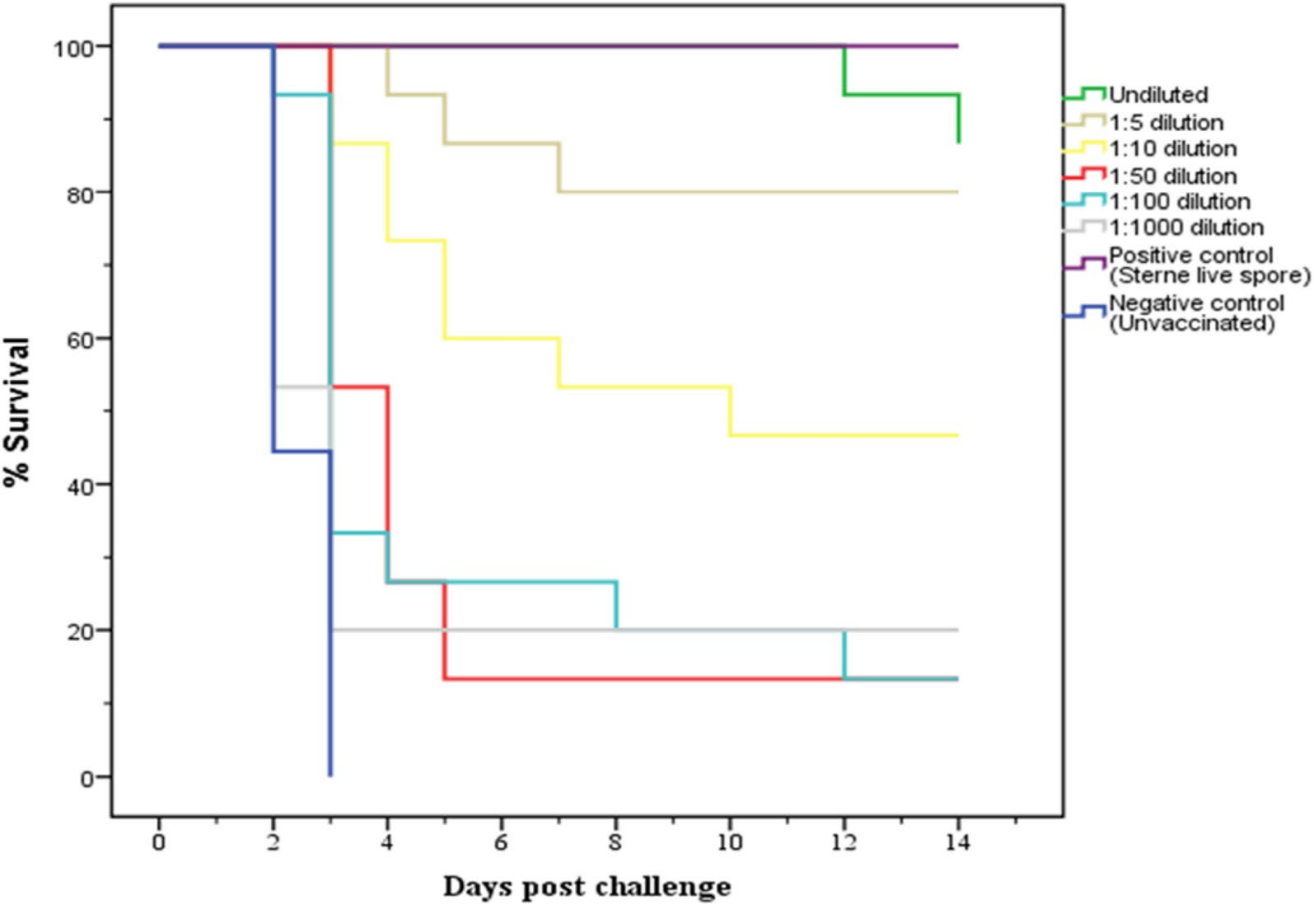
**Kaplan–Meier plots indicating the survival of A/J mice after passive transfer of naïve and immune goat sera at different dilutions.** For each group (undiluted and diluted), 500 µL of serum from each goat subject (*n* = 5) were inoculated into three A/J mice (*n* = 15 for each group). Positive control mice (*n* = 12, three A/J mice per goat) received undiluted sera from immune goats (*n* = 4) that survived a virulent *B. anthracis* challenge. Negative control mice (*n* = 9, three A/J mice per goat) received undiluted sera from naïve goats (*n* = 3). The mice were lethally challenged after 24 h with 1.92 × 10^5^ spores per dose from the Sterne 34F2 vaccine strain.

A total of 75 A/J mice were passively immunized with undiluted and diluted immune sera ranging from 1:1000, 1:100, 1:50, 1:10 and 1:5 collected at week 17 from individual goats immunized with Sterne live spore vaccine. The protective effect of the immune sera was assessed following challenge with *B. anthracis* 34F2 spores. The level of protection of the immune goat sera was dependent on the dilution used, while sera from the naïve goats failed to protect mice (Figure 3). All nine A/J mice that received negative (naive) goat sera died within 3 days of challenge, whereas all mice (12/12) that received serum from the positive controls (Sterne-immunized goats surviving lethal anthrax spores challenge) survived for 14 days (Figure 3).

Mice receiving immune goat sera at higher dilutions showed lower survival than those receiving sera at lower dilutions. Analysis of the survival curves showed no difference between the negative control and the 1:1000 diluted sera group (*p* = 0.290). However, survival curves from sera groups diluted 1:100 or less were significantly different from the negative control (*p* ≤ 0.004). For the undiluted immune goat sera, 13 of the 15 (87%) mice survived the challenge and was no different from the positive control group (*p* = 0.165). Two mice injected with undiluted sera from vaccinated goats D6 and D20 only died on day 12 (euthanized) and 14, respectively (Table 1). Both mice were confirmed positive for anthrax by isolation of *B. anthracis* from the spleen and liver. Mice that received undiluted immune serum had a mean survival of 13.9 days compared to the mice that received different dilutions for which survival ranged between 5 and 12 days depending on the dilution (Table 1). The 1:5, 1:10, 1:50, 1:100 and 1:1000 dilutions had 80, 47, 13, 13 and 20% survival rates respectively with most of the mice dying within the first 5 days (Figure 3; Table 1). There was no difference in survival times between 1:5 and the positive control groups (*p* ≥ 0.0829). However, dilution of immune sera by 1:10 or more afforded significantly less protection to the mice when compared to the positive control (*p* ≤ 0.002).

**Table 1:** **Passive protection test results of A/J mice.**

Goat serum			Survival (Time to death^b^)					
Animal	PA-ELISA (week 0)	TNA (week 0)	Undiluted sera	1:5	1:10	1:50	1:100	1:1000
D5^a^	2680 (212)	3410 (0)	3/3	3/3	2/3 (11)	0/3 (4, 4, 4)	1/3 (3, 3)	0/3 (2, 2, 2)
D6^a^	4190 (380)	1800 (0)	2/3 (12)	3/3	2/3 (5)	0/3 (3, 3, 3)	0/3 (2, 3, 3)	1/3 (2, 3)
D20^a^	8050 (310)	2530 (0)	2/3 (14)	3/3	1/3 (4, 7)	1/3 (3, 5)	0/3 (3, 3, 12)	0/3 (2, 2, 3)
D29^a^	7510 (240)	2280 (0)	3/3	2/3 (5)	2/3 (10)	1/3 (3, 5)	1/3 (3, 8)	1/3 (3, 3)
D31^a^	1450 (220)	240 (0)	3/3	1/3 (4, 7)	0/3 (3, 3, 4)	0/3 (3, 3, 4)	0/3 (3, 3, 4)	1/3 (2, 3)
Negative control^ac^								
D13	590 (80)	0 (0)	0/3 (2, 2, 2)					
D39	100 (130)	0 (0)	0/3 (3, 2, 3)					
D77	120 (90)	0 (0)	0/3 (3, 3, 2)					
Positive control^d^								
8175	39 590 (212)	5870 (0)	3/3					
8182	17 000 (0)	7300 (0)	3/3					
8210	185 500 (173)	17 380 (0)	3/3					
8212	1 223 430 (405)	35 720 (0)	3/3					

^a^Sera collected at 17 weeks

^b^Time to death (in days) of non-surviving mice; Mice injected with undiluted and diluted hyper-immune goat sera and challenged with spores from the Sterne 34F2 vaccine strain 24 h later and immune sera were diluted from 1:5 to 1:1000

^c^Sera collected from goats (*n* = 3) receiving sterile saline only; all mice died within 3 days of challenge with *B. anthracis* Sterne strain

^d^Sera sourced from goats (*n* = 4) surviving virulent anthrax spores after two vaccinations with the Sterne live spore vaccine in a previous study [12]

### Comparison of ELISA, TNA and passive protection test

The ELISA and TNA titres of sera from individual goats at week 17 (after second vaccine dose) showed no correlation (r_s_ = 0.404; *p* < 0.135). PA antibody measured in sera from individual vaccinated goats did not correlate with passive protection in A/J mice using either diluted or undiluted sera (r_s_ ≤ 0.487; *p* ≥ 0.066). Significant positive correlations between toxin neutralizing antibodies and protection of A/J mice were observed following passive transfer of immune goat sera diluted at 1:5 and 1:10 respectively (Table 2).

**Table 2:** **Pearson correlation between toxin neutralizing titres (goat serum) and mouse survival.**

	Undiluted sera	1:5	1:10	1:50	1:100	1:1000
Pearson correlation (r_s_)	0.064	0.522	0.655	0.203	0.324	−0.317
Significance (2-tailed)	0.820	0.046	0.008	0.469	0.238	0.249

Toxin neutralization titres to survival in A/J mice following passive transfer of various dilutions of immune goat sera before challenge with spores from Sterne 34F2 vaccine strain. Correlation analysis was performed for each dilution using individual TNA titres and survival

## Discussion

Results of this study demonstrate a potential protective capacity of the humoral response elicited in Sterne live spore vaccinated goats using the passive protection test in A/J mice. The passive mouse protection model can be effectively used to evaluate the immune response in livestock animals. A/J-mice were utilized because of their known susceptibility to toxigenic but non-encapsulated strains of *B. anthracis* such as Sterne or STI-1 [19], caused by a deficiency of C5 in the complement system [14]. A/J mice react in a dose-dependent manner unlike CBA/J and BALB/c mice to a challenge with those vaccine strains. Passively transferred goat immune sera protected A/J mice from lethal challenge with protection correlated to sera dilution levels. We observed that sera from twice immunized goats (goats vaccinated twice with Sterne live spore vaccine 12 weeks apart) with neutralizing antibody titres ≥ 200 afforded robust protection against 1.92 × 10^5^ *B. anthracis* Sterne strain spores challenge in the A/J mice model (Table 1). The number of goats (*n* = 5) as well as mice (*n* = 3 per assessed serum dilution) used in this study limited statistical power and results should be interpreted in this light. As the main focus was to investigate protection only the PA-ELISA IgG antibody titres in vaccinated and non-vaccinated goats were measured, but IgM antibody response can be investigated in future. However, this study provides valuable insight in the potential use of immune assays and the passive protection test to measure presumptive protection conferred following vaccination with anthrax vaccines.

The *B. anthracis* TNA is a technique designed to measure the ability of toxin neutralizing antibody to protect certain susceptible cell lines from the lethal effects of anthrax toxin [20, 21]. TNA is not species dependent and has been developed for use with multiple species [18]. Here, the TNA titres directly correlated to survival in mice with 1:5 and 1:10 diluted immune goat sera in the passive protection test (Table 1). The inability to show correlation using undiluted sera was likely due to the almost uniform survival observed following challenge, with the two non-survivors protected for 12 and 14 days respectively (Table 1). We noticed that TNA titres of just over 200 can confer protection in the passive protection test performed in this study. Accordingly serum from goat D31 provided 100% (3/3) protection to A/J mice for undiluted sera (Table 1) despite a relatively low TNA antibody level but did not provide protection when diluted 1:10; 1:50 and 1:100. This is in line with data from guinea pig studies. Reuveny et al. [20] showed that TNA titres as low as 220 will provide some level of protection. Toxin neutralization titres were absent to low, 4 weeks after the first vaccination but were evident at week 17. This supports the results published by Ndumnego et al. [12] who showed a strong increase in titres after a second vaccination of goats with the same vaccine (34F2).

Our study provides evidence that the mouse protection model can be effectively used to evaluate the immune response of target animals like livestock, thereby eliminating the ethical and logistical challenges associated with large animal trials. It also obviates the need of fully virulent *B. anthracis* strains for the challenge and consequent biosafety level specification. This test would be beneficial to assess presumptive protective capacities of new vaccine formulations against anthrax in animals prohibitive to be experimentally challenged (valuable livestock, endangered/critical wildlife populations). Moreover, it could be used to evaluate and monitor the presumptive protective capacity of vaccination procedures currently in veterinary use, e.g. to determine the herd immunity after a vaccination campaign. In order to unambiguously proof the significance of the correlation between the passive mouse protection test and TNA titres as an immunological correlate for protection in various animal species more such data sets are needed. Then, one could imagine that in the future TNA titres could serve as the correlate for protection also for commercial vaccine production, making the animal challenge tests for vaccine batches obsolete.

Certainly, the final proof for the correlation of TNA titres and protection would need appropriate lethal challenge experiments with various animal species. As this approach is, correctly, considered unethical such data will have to be collected from further small scale experiments and, where possible, from epidemiological data of the veterinary field usage of commercial vaccines. For the latter it would be helpful to monitor by TNA the actual immune responses in various target animals.

## Abbreviations

*B. anthracis*: *Bacillus anthracis*
PA: protective antigen
ELISA: enzyme-linked immunosorbent assay
TNA: toxin neutralization assay
LF: lethal factor
EF: edema factor
LT: lethal toxin
ET: edema toxin
OBP: Onderstepoort Biological Products
LD: lethal dose
OD: optical density
MTT: 3-(4.5-dimethylthiazol-2-yl)-2.5-diphenyltetrazolium bromide
